# Supplementary material for: Deletion of wfs1 Impairs Oligodendrocyte Precursor Cells Dorsal Distribution and Myelination Through the wfs1-hmgcs1 Axis in Zebrafish
Source: Biology (Basel). 2026 Aug 16;15(16):1402. doi: 10.3390/biology15161402 (PMC13510151; doi:10.3390/biology15161402)
Supplement: Supplementary file 1 [file biology-15-01402-s001.zip › File S1-Transcriptome sequencing data and differentially expressed genes (DEGs) in wfs1-- zebrafish at 5 dpf/2.KEGG/wfs1KOvsWT/DOWN/src/dre00020.html]

dre00020


- dre:368262

- Down regulated genes

368262(-1.10235845855677)

- dre:550579

- Down regulated genes

550579(-8.88438771541115)

- dre:322670

- Down regulated genes

322670\_1(-2.18297697354538)

- dre:322670

- Down regulated genes

322670\_1(-2.18297697354538)

Close
